# Parry-Romberg Syndrome: A Rare Case with Diagnostic Challenges and Orthodontic Implications

**DOI:** 10.7759/cureus.93225

**Published:** 2025-09-25

**Authors:** Shubham Patel, P. Narayana Prasad, Tarun Sharma, Anupa Rawat, Amit Agarwal

**Affiliations:** 1 Department of Orthodontics, Seema Dental College and Hospital, Rishikesh, IND; 2 Department of Oral and Maxillofacial Surgery, Seema Dental College and Hospital, Rishikesh, IND

**Keywords:** facial asymmetry, orthodontic management, parry-romberg, parry-romberg syndrome, progressive hemifacial atrophy, rare case

## Abstract

Parry-Romberg syndrome (PRS) is a rare acquired disorder characterized by progressive hemifacial atrophy involving the skin, subcutaneous tissue, muscles, cartilage, and bone. The disease usually progresses for two to 20 years before stabilizing, with extracranial involvement of the trunk and extremities occasionally reported. The etiology remains uncertain, with proposed immunological, genetic, and neurovascular mechanisms. We present the case of a 24-year-old female with PRS who exhibited intraoral and extraoral soft tissue atrophy, localized alopecia, ocular changes, and otologic involvement. Intraoral examination revealed delayed dental development and an anterior crossbite on the unaffected side. Despite marked craniofacial asymmetry, no neurological deficits were identified. The patient is currently receiving orthodontic treatment to address occlusion and improve facial balance. This case highlights the importance of early recognition and multidisciplinary management of PRS, where orthodontic intervention contributes significantly to functional and psychosocial outcomes. Further studies are needed to clarify the long-term effects of orthodontic and surgical rehabilitation in this rare condition.

## Introduction

Parry-Romberg syndrome (PRS), also known as progressive hemifacial atrophy (PHA), is a rare condition characterized by progressive wasting of the facial skin and soft tissues, occasionally extending to muscle, cartilage, and bone [1,2]. First described by Parry in 1825 and Romberg in 1846, with the term “progressive hemifacial atrophy” introduced by Eulenberg in 1871 [3-5], the condition remains uncommon, with only a few hundred cases reported worldwide [6].

PRS typically begins in childhood or adolescence and progresses for two to 20 years before stabilizing [1,6,7]. Although usually unilateral, involvement may extend to the extremities or trunk [2,8,9]. Most cases are sporadic, though rare familial clustering suggests a possible genetic contribution [10-12]. Proposed etiologies include autoimmune, neurovascular, infectious, traumatic, and genetic mechanisms, but no single explanation has been confirmed [6,7,10].

Although the dermatological, neurological, and ophthalmological features of PRS are well documented, oral and orthodontic manifestations remain comparatively underreported, despite their significant impact on function and aesthetics. This report emphasizes these overlooked aspects by describing a 24-year-old female patient with PRS who presented with unilateral anterior crossbite and delayed dental development and by discussing the orthodontic perspective within a multidisciplinary framework.

## Case presentation

A 24-year-old female patient was referred to the Department of Orthodontics and Dentofacial Orthopaedics, Seema Dental College and Hospital, Rishikesh, Uttarakhand, India, with the chief complaint of facial deformity and forwardly placed lower anterior teeth associated with an unesthetic smile. The patient reported a history of progressive atrophy on the right side of the face, which began around the age of six years. The deformity became more apparent by age 13 and continued to progress until approximately 20 years of age, after which the condition appeared to stabilize.

**Figure 1:**
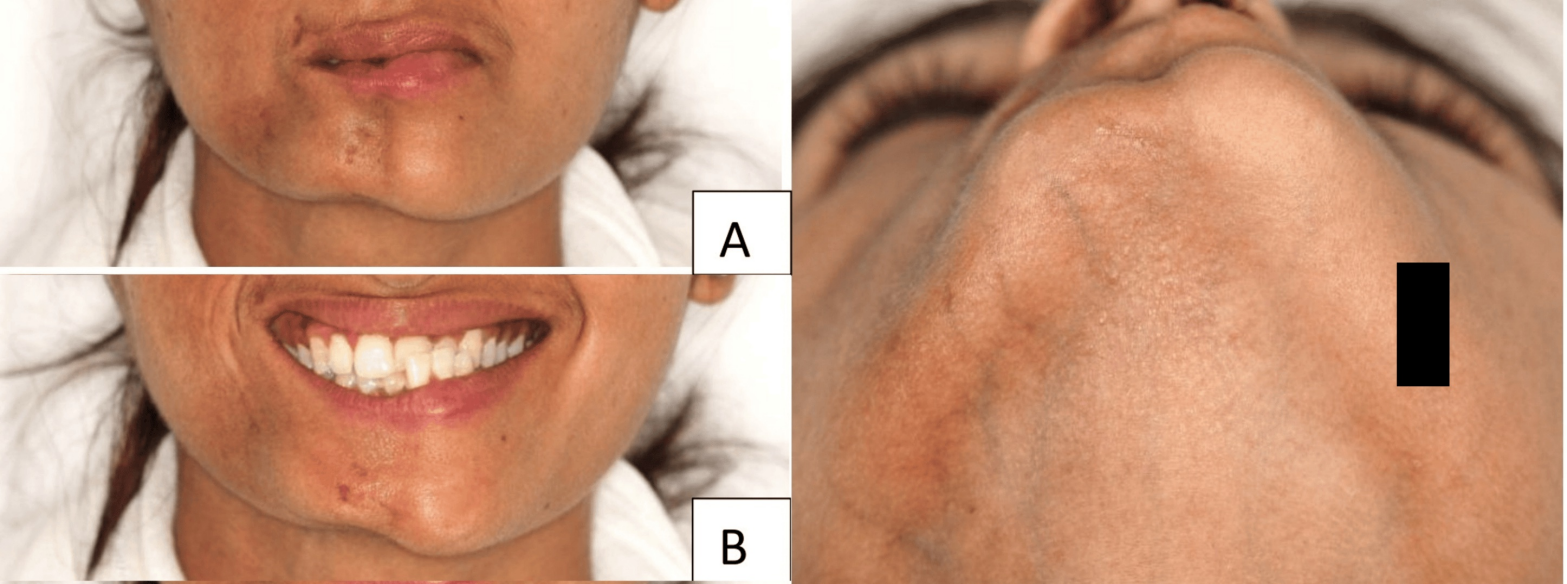
Extraoral clinical photographs of the patient (A) Frontal view showing right-sided hemifacial atrophy with lip deviation, hyperpigmentation, and a scar-like depression in the symphyseal region (coup de sabre); (B) Smiling view demonstrating exaggerated shrinkage of the skin on the affected side, consistent with subcutaneous fat and soft tissue loss; (C) Worm’s-eye view highlighting prominent superficial veins due to thinning of overlying soft tissues.

On extraoral examination, marked facial asymmetry was observed, with hypoplasia of the right side of the face and deviation of the lip toward the affected side, resulting in lip incompetence. Areas of hyperpigmentation were observed along with a scar-like depression in the symphyseal region, consistent with coup de sabre. Prominent veins were visible on the right side, attributed to loss of subcutaneous fat and thinning of the overlying soft tissues. The asymmetry became more pronounced on smiling, when the affected side exhibited exaggerated skin shrinkage, a feature indicative of subcutaneous fat loss and underlying soft tissue deficiency (Figures 1A-1C).

**Figure 2:**
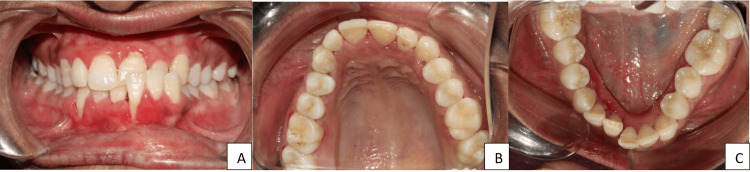
Intraoral clinical photographs of the patient (A) Frontal view showing mild anterior crowding in both arches, anterior crossbite on the left side, and gingival recession in the lower anterior region; (B) Maxillary occlusal view demonstrating arch asymmetry with constriction of the right side; (C) Mandibular occlusal view revealing anterior crowding and asymmetry of the dental arch.

Intraoral evaluation revealed mild anterior crowding in both arches, an anterior crossbite on the left side, and gingival recession in the lower anterior region. Both maxillary and mandibular arches showed asymmetry with marked constriction of the right side. The American Board of Orthodontics (ABO) Discrepancy Index [13] confirmed an Class III molar relationship (Figures 2A-2C).

**Figure 3:**
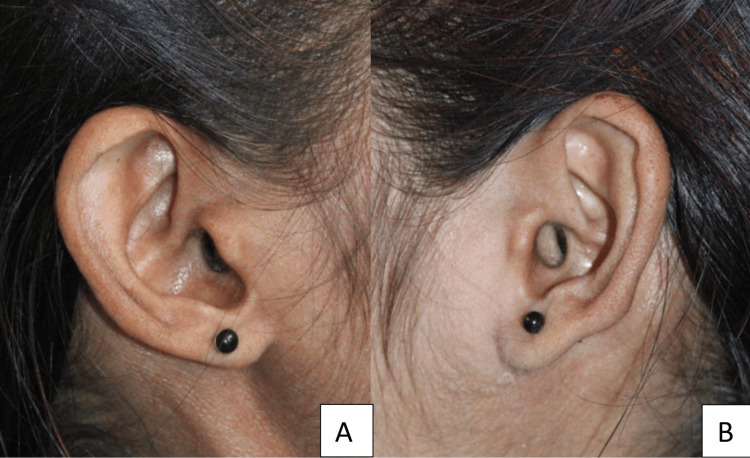
Ear morphology of the patient (A) Right ear showing reduced size and abnormal shape, consistent with soft tissue hypoplasia; (B) Comparison with the contralateral ear, which appears normal in morphology and size.

A review of family history through pedigree charting and genetic counseling did not reveal any hereditary component, supporting the diagnosis of an isolated case. General clinical findings included muscle wasting on the right side of the face, deviation of the nose, and prominent superficial veins. The right ear was misshapen and reduced in size (Figures 3A-3B).

**Figure 4:**
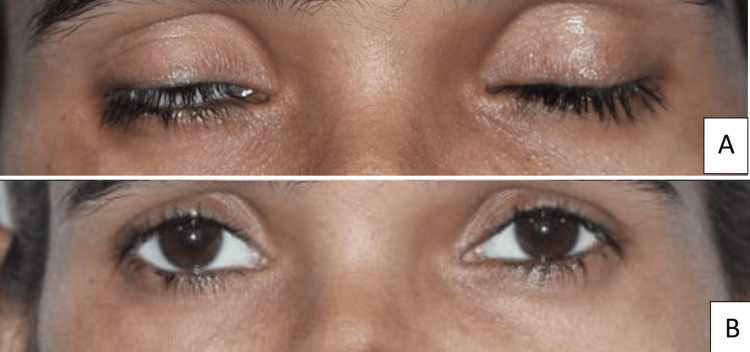
Ocular findings of the patient (A) Frontal view showing lagophthalmos (noted in the right eye, reflecting impaired eyelid closure due to soft tissue and fat atrophy around the orbit, a recognized manifestation of Parry-Romberg syndrome) and loss of periorbital fat; (B) Close-up demonstrating an interpupillary cant on the right side.

Ocular changes were also present, including enophthalmos, loss of periorbital fat, lagophthalmos, and an interpupillary cant (Figures 4A-4B).

**Figure 5:**
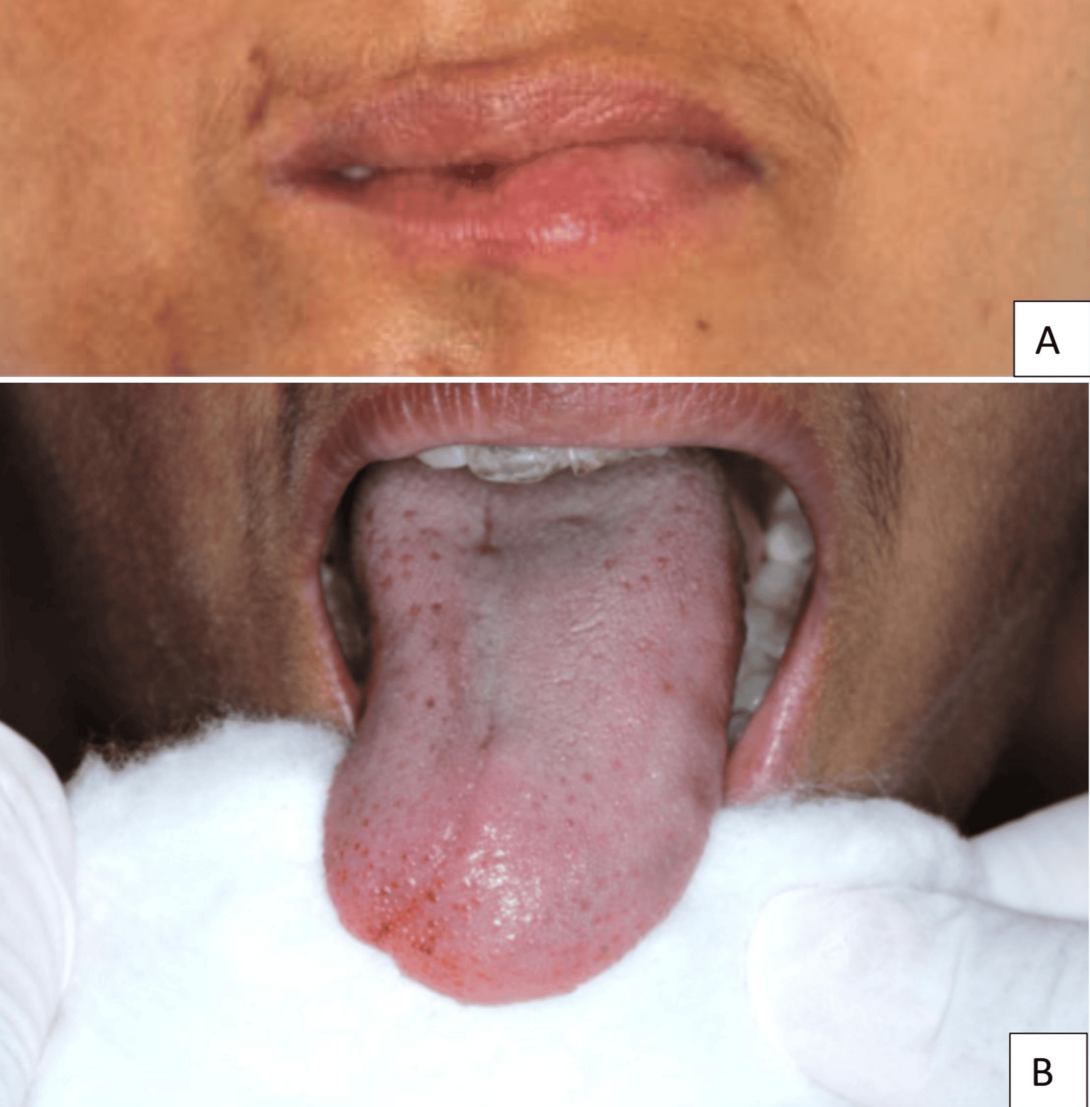
Intraoral soft tissue changes (A) Lip atrophy evident on the right side; (B) Tongue atrophy with reduced bulk and deviation toward the right side, consistent with soft tissue and muscular involvement.

Lip and tongue atrophy were additionally noted (Figures 5A-5B).

**Figure 6:**
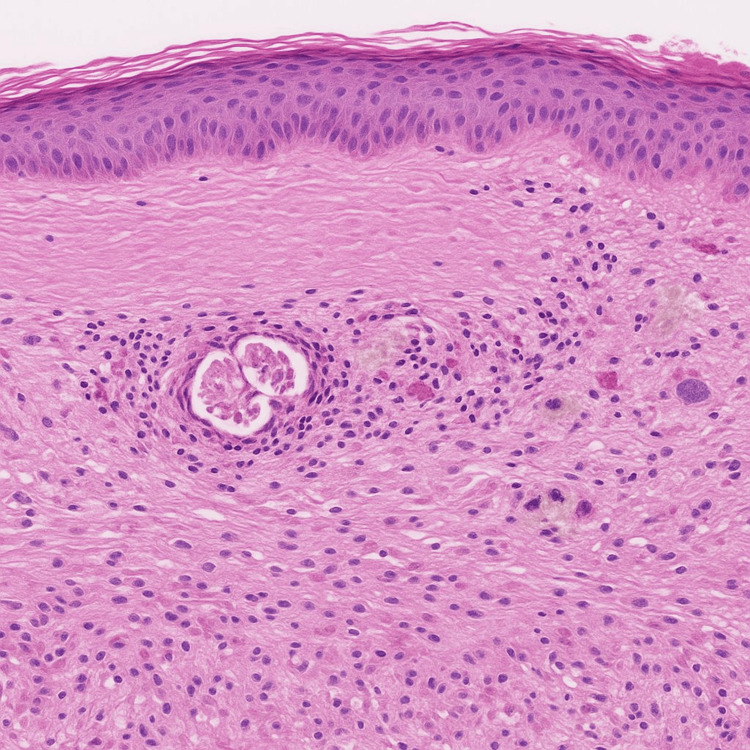
Histopathological examination of the affected skin Atrophic epidermis, mild sclerosis of the upper dermis, loss of fat spaces surrounding eccrine glands (explaining hypohidrosis), mild perivascular lymphocytic infiltrate, and focal pigment incontinence (Hematoxylin and Eosin stain, ×100).

Histopathological examination of a skin biopsy demonstrated an atrophic epidermis, mild sclerosis of the upper dermis, and loss of fat spaces surrounding eccrine glands, a finding that explains the presence of hypohidrosis. Mild perivascular lymphocytic infiltrate and focal pigment incontinence were also noted. These features were consistent with Parry-Romberg syndrome and distinguished the condition from localized scleroderma (morphea), which typically demonstrates more pronounced dermal fibrosis (Figure 6).

**Figure 7:**
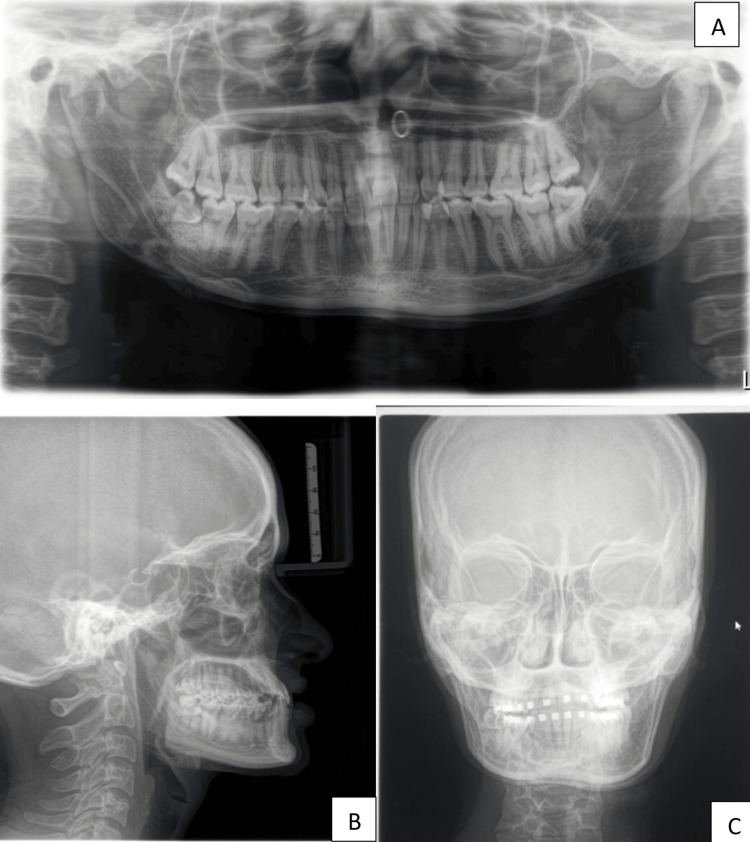
Radiographic evaluation of the patient (A) Orthopantomogram showing reduced ramus and corpus length on the right side along with an impacted third molar; (B) Lateral cephalogram demonstrating a skeletal Class III jaw relationship with a concave soft tissue profile (SNA=82°, SNB=86°, ANB=–4°); (C) Posteroanterior cephalogram confirms facial asymmetry. SNB: Sella-Nasion-Point B angle; ANB: Point A-Nasion-Point B angle.

Radiographic investigations further supported the diagnosis. An orthopantomogram (OPG) showed reduced ramus and corpus length on the right side along with an impacted third molar. The patient also reported delayed eruption of teeth on the affected side. Cephalometric analysis revealed a skeletal Class III jaw relationship with a concave soft tissue profile (S=Sella, N=Nasion, A=Point A (subspinale), B=Point B (supramentale); SNA= 82°, SNB= 86°, ANB= -4°) and an increased mandibular base length compared with the maxilla (1.8:2.2). A posteroanterior cephalogram confirmed facial asymmetry (Figures 7A-7C).

**Figure 8:**
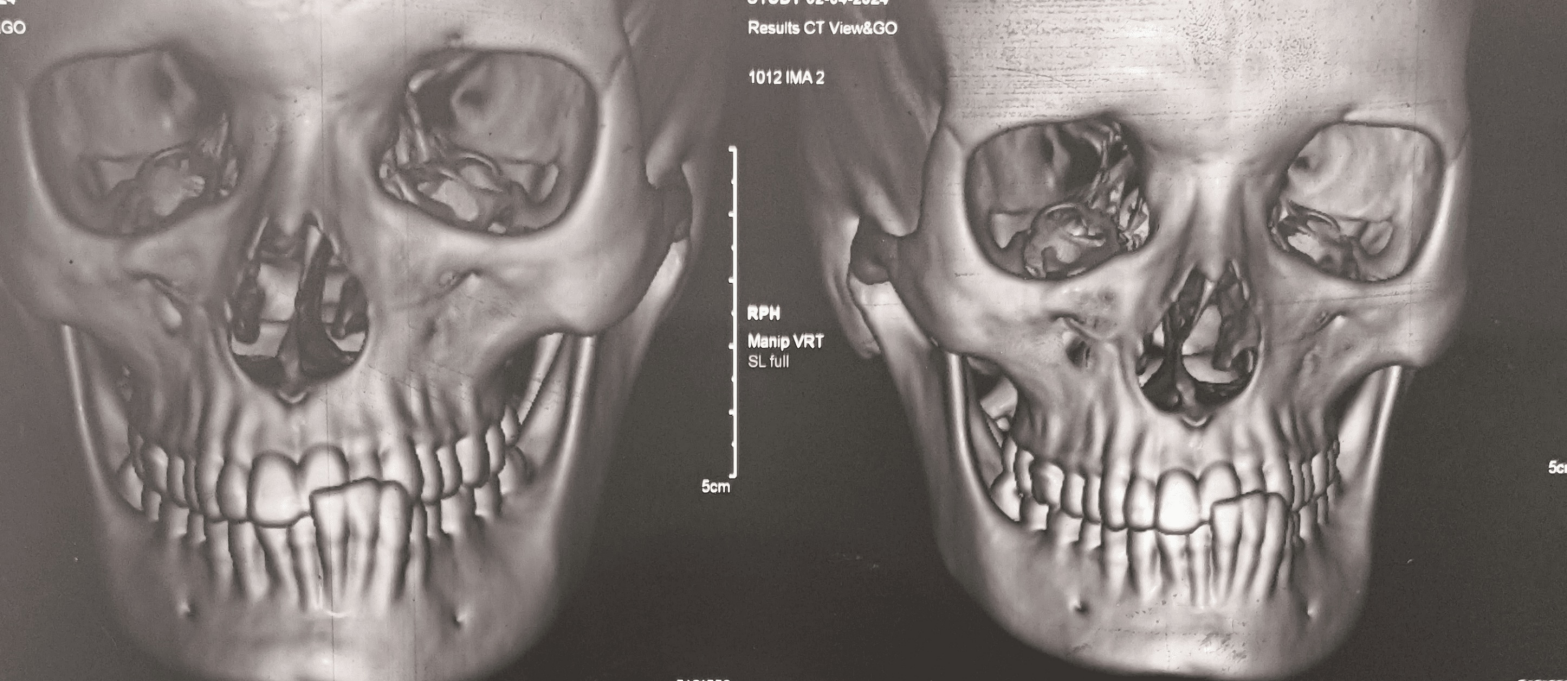
Computed tomography (CT) of the craniofacial skeleton CT imaging demonstrating atrophy of the right zygomatic, maxillary, and mandibular bones, with inferior positioning of the right orbital floor compared to the left.

Computed tomography demonstrated atrophy of the right zygomatic, maxillary, and mandibular bones, with the right orbital floor positioned inferiorly relative to the left (Figure 8).

Taken together, the clinical, radiographic, and histopathological findings established a diagnosis of Parry-Romberg syndrome associated with skeletal Class III and dentoalveolar Angle’s Class III malocclusion.

**Figure 9:**
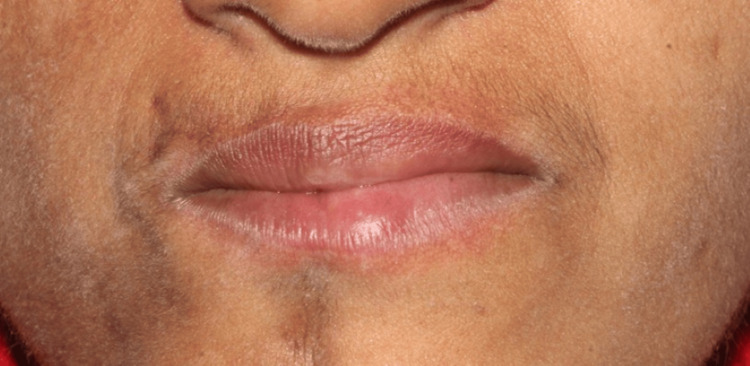
Functional improvement of the lips The close-up shows restoration of lip competency and reduction of deviation following six months of treatment.

**Figure 10:**
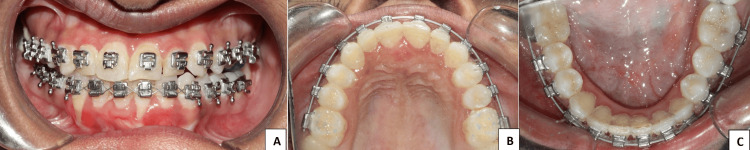
Treatment progress following orthodontic management (A–C) Intraoral views showing arch alignment and correction of the anterior crossbite after six months of fixed orthodontic mechanotherapy.

As the patient’s growth was complete and disease progression had been static for the preceding four years, a multidisciplinary treatment plan was developed. Orthodontic intervention included establishing a facebow relation to assess centric relation versus centric occlusion, followed by fixed orthodontic mechanotherapy using MBT 0.022″ slot appliances (MBT™ Versatile+ Appliance System, 3M, MN, USA) and other appliances like self-ligating to align the arches, correct the crossbite, and improve occlusion. In consultation with plastic surgery, restorative interventions such as autologous fat grafts or silicone implants were recommended to address soft tissue deficits, with aesthetic reconstruction of the right hemiface planned for subsequent stages. Hyaluronic acid fillers were considered as a minimally invasive alternative for soft tissue augmentation. The patient has been under active orthodontic management for six months, and visible improvements have already been achieved (Figures 9, 10A-10C).

For clarity, the key clinical, radiographic, and histopathological findings of this patient are consolidated in Table 1, providing a concise overview of the case.

**Table 1:** Summary of clinical, radiographic, and histopathological findings in the case OPG: Orthopantomogram; SNA: Sella-Nasion-Point A angle; SNB: Sella-Nasion-Point B angle; ANB: Point A-Nasion-Point B angle; CT: Computed Tomography; PRS: Parry-Romberg Syndrome

Domain	Findings
Patient demographics	24-year-old female patient; growth complete; isolated case (no family history)
Extraoral features	Right-side facial hypoplasia; lip deviation and incompetence; nasal deviation; prominent superficial veins; small misshapen right ear; ocular changes (enophthalmos, loss of periorbital fat, lagophthalmos, interpupillary cant)
Intraoral findings	Mild anterior crowding (both arches); left-sided anterior crossbite; gingival recession (lower anterior); arch constriction on right side; end-on Class III molar relationship
Radiographic findings	OPG: shorter ramus & corpus on right, impacted 3rd molar; delayed eruption on right; Cephalometric: skeletal Class III (SNA=82°, SNB=86°, ANB = –4°), concave profile; CT: bone atrophy (zygomatic, maxillary, mandibular), inferior displacement of orbital floor on right
Soft tissue and orthodontic features	Lip and tongue atrophy; thin buccal soft tissue; dentoalveolar Angle’s Class III malocclusion; anterior crossbite; arch asymmetry
Histopathology	Atrophic epidermis; mild sclerosis of upper dermis; loss of fat around eccrine glands (hypohidrosis); mild perivascular lymphocytic infiltrate; focal pigment incontinence — consistent with Parry-Romberg syndrome (PRS)
Diagnosis	PRS with skeletal Class III and dentoalveolar Angle’s Class III malocclusion

## Discussion

PRS, also known as progressive facial hemiatrophy (PFH), is a rare and variant form of craniofacial atrophy characterized by unilateral facial involvement and, less commonly, extension to the trunk and limbs [1]. Classical descriptions include not only soft-tissue atrophy but also ocular and hair changes, trigeminal neuropathy, and contralateral Jacksonian seizures in some instances [14]. The disease typically progresses over a prolonged period, often two to 20 years, before reaching stabilization [1,6].

There is an ongoing debate regarding whether PRS and localized scleroderma (en coup de sabre) belong to the same disease spectrum, given their overlapping clinical and histopathological features [15]. However, their distinct patterns of tissue involvement and inflammation suggest that while they share some characteristics, they remain separate entities for now.

**Table 2:** Comparative features of Parry-Romberg syndrome and localized scleroderma (morphea)

Feature	Parry-Romberg Syndrome (PRS)
Onset	Childhood or adolescence; progressive course 2–20 years before stabilization
Laterality	Predominantly unilateral, affecting one side of the face
Skin changes	Progressive hemifacial atrophy with hyperpigmentation, “coup de sabre” scar, loss of subcutaneous tissue
Subcutaneous tissue	Marked loss of fat, muscle wasting, deeper atrophy
Neurological/Ocular	May involve trigeminal neuralgia, seizures, enophthalmos, and ocular abnormalities
Oral/Orthodontic	Delayed dental development, malocclusion, jaw asymmetry, tongue/lip atrophy
Histopathology	Atrophic epidermis, fat loss, mild dermal sclerosis, perivascular lymphocytes

The etiology of PRS remains elusive. Some researchers implicate neurovasculopathy, whereas imaging studies have revealed cerebral microhemorrhages, suggesting vascular compromise as a contributing factor [16]. Cory et al. proposed a mechanism involving inflammatory damage to the cervical sympathetic ganglia and carotid plexus, leading to sympathetic dysfunction and craniofacial developmental issues, correlating with neurological symptoms observed in pediatric cases [17]. Other proposed etiologies include autoimmune dysregulation, genetic predispositions (with occasional familial clustering), trauma, and infectious triggers [6,7,10].

The hallmark clinical presentation of PRS is progressive atrophy of the skin, subcutaneous fat, and occasionally muscle and bone, resulting in a striking facial asymmetry [2,6]. One of the earliest clinical signs is often a linear midline facial cleft or indentation, sometimes accompanied by hyperpigmentation or fibrotic changes of the skin [6]. Alopecia, along with loss of facial hair in the affected region, is also frequently observed [18]. Oral manifestations include lip and tongue atrophy, unilateral tooth exposure, delayed eruption, and root hypoplasia. Jaw hypoplasia and arch constriction may lead to dental malocclusion and compromised function; extraction sites on the affected side frequently demonstrate impaired bone healing [19].

Neurologically, PRS may present with trigeminal neuralgia, facial paresthesia, migraines, or focal epilepsy. Neuroimaging may reveal ipsilateral intracranial calcifications, white-matter hypodensities, or even vascular anomalies such as aneurysms. Electroencephalography is often nonspecific [14,17,20]. Interestingly, our patient did not exhibit any neurological symptoms, highlighting the heterogeneous nature of PRS.

Ophthalmologic involvement often includes enophthalmos due to loss of periorbital fat. Although rare, some cases have reported retinal vasculitis or uveitis [21]. Additionally, otologic anomalies such as small ear size on the affected side may be visible even in the absence of auditory dysfunction.

Histopathological examination is crucial for distinguishing PRS from morphea. PRS typically shows epidermal thinning, dermal atrophy, loss of subcutaneous fat, and adnexal structure degeneration, accompanied by mild perivascular lymphocytic infiltration. Notably, elastic fibers are preserved, a feature that contrasts with the dense dermal fibrosis, collagen homogenization, and elastic fiber loss seen in localized scleroderma [15]. When Lyme disease is a differential consideration, silver staining may help rule in or out Borrelia burgdorferi infection [7,15].

Diagnosis is primarily clinical, supported by histopathology and imaging. Disease severity is often determined by the age of onset and anatomical extent but does not always correspond to the degree of neurological involvement [1,10,17].

Currently, there is no cure for PRS. In the active phase, clinicians may trial corticosteroids or immunosuppressive medications empirically to attenuate progression, though robust evidence is lacking [7,15]. Once the disease stabilizes, orthodontic interventions are essential to address malocclusion, arch constriction, and functional disruption, especially when provided early. Reconstructive surgery, including grafting, flap procedures, or implant placement, plays a pivotal role in restoring facial symmetry [9,19,21]. In the present case, six months of orthodontic treatment has already produced notable improvements in the adjacent soft tissues, underscoring the value of early therapeutic intervention.

## Conclusions

PRS is a rare craniofacial disorder of unknown etiology, where early diagnosis and timely intervention are crucial to optimize the quality of life. In this case, diagnosis at age 24 allowed six months of orthodontic therapy, which resulted in measurable improvements in occlusal balance, lip competence, and adjacent soft tissues. Earlier recognition might have limited the progression of facial deformity during growth. Future research should focus on the safety, timing, and long-term effectiveness of orthodontic, surgical, and multidisciplinary interventions, as well as their impact on both functional and psychosocial outcomes.
